# An engineered pathway for glyoxylate metabolism in tobacco plants aimed to avoid the release of ammonia in photorespiration

**DOI:** 10.1186/1472-6750-11-111

**Published:** 2011-11-21

**Authors:** Josirley de FC Carvalho, Pippa J Madgwick, Stephen J Powers, Alfred J Keys, Peter J Lea, Martin AJ Parry

**Affiliations:** 1 Embrapa Soybean, Londrina, Paraná, Brazil, Rodovia Carlos Strass, Distrito da Warta; C.P.: 6001; 86001-970; Londrina - PR - Brasil; 2 Rothamsted Research, Harpenden, Hertfordshire, AL5 2 JQ, UK; 3 Lancaster Environment Centre, Lancaster University, Biological Sciences, Lancaster, LA1 4YQ, UK

## Abstract

**Background:**

The photorespiratory nitrogen cycle in C_3 _plants involves an extensive diversion of carbon and nitrogen away from the direct pathways of assimilation. The liberated ammonia is re-assimilated, but up to 25% of the carbon may be released into the atmosphere as CO_2_. Because of the loss of CO_2 _and high energy costs, there has been considerable interest in attempts to decrease the flux through the cycle in C_3 _plants. Transgenic tobacco plants were generated that contained the genes *gcl *and *hyi *from *E. coli *encoding glyoxylate carboligase (EC 4.1.1.47) and hydroxypyruvate isomerase (EC 5.3.1.22) respectively, targeted to the peroxisomes. It was presumed that the two enzymes could work together and compete with the aminotransferases that convert glyoxylate to glycine, thus avoiding ammonia production in the photorespiratory nitrogen cycle.

**Results:**

When grown in ambient air, but not in elevated CO_2_, the transgenic tobacco lines had a distinctive phenotype of necrotic lesions on the leaves. Three of the six lines chosen for a detailed study contained single copies of the *gcl *gene, two contained single copies of both the *gcl *and *hyi *genes and one line contained multiple copies of both *gcl *and *hyi *genes. The gcl protein was detected in the five transgenic lines containing single copies of the *gcl *gene but hyi protein was not detected in any of the transgenic lines. The content of soluble amino acids including glycine and serine, was generally increased in the transgenic lines growing in air, when compared to the wild type. The content of soluble sugars, glucose, fructose and sucrose in the shoot was decreased in transgenic lines growing in air, consistent with decreased carbon assimilation.

**Conclusions:**

Tobacco plants have been generated that produce bacterial glyoxylate carboligase but not hydroxypyruvate isomerase. The transgenic plants exhibit a stress response when exposed to air, suggesting that some glyoxylate is diverted away from conversion to glycine in a deleterious short-circuit of the photorespiratory nitrogen cycle. This diversion in metabolism gave rise to increased concentrations of amino acids, in particular glutamine and asparagine in the leaves and a decrease of soluble sugars.

## Background

**Figure 1:**
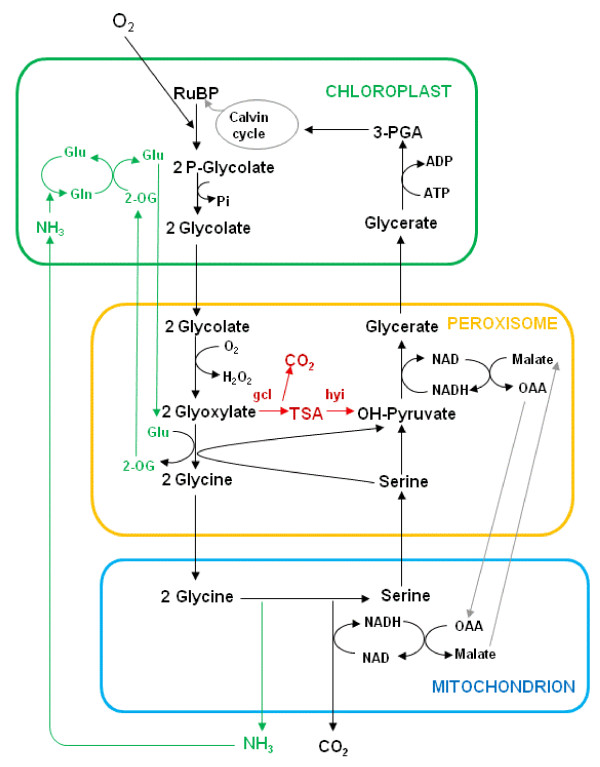
**The Photorespiratory Nitrogen Cycle showing the alternative route through tartronic semialdehyde**. The ammonia produced in the conversion of glycine to serine passes out of the mitochondrion and is reassimilated (green pathway). The CO_2 _released in the mitochondrion escapes to the intercellular spaces. The red pathway represents the intended short-circuit in the photorespiratory cycle by the bacterial enzymes gcl and hyi. Figure adapted from Wingler et al. [39] and Keys et al. [1]. (Glu: Glutamate; Gln: Glutamine; 2OG: 2-oxoglutarate; OAA: oxaloacetate; TSA tartronic semialdehyde).

The photorespiratory nitrogen cycle in C_3 _plants involves a considerable diversion of carbon and nitrogen away from the direct pathways of assimilation [1,2]. Ultimately all of the nitrogen is re-assimilated, but up to 25% of the carbon may be released back to the atmosphere as CO_2 _and both of these wasteful processes consume substantial amounts of energy [3-5]. High rates of photorespiration have been detected in C_3 _plants and there is now growing evidence that photorespiration also takes place in C_4 _plants, although to a much lesser degree [6-9]. An unusual feature of the photorespiratory nitrogen cycle is that it requires the action of enzymes and transporters located in three different subcellular compartments, the choloroplasts, peroxisomes and mitochondria, and also possibly the cytoplasm (Figure 1).

The key enzyme responsible for photosynthetic carbon assimilation is ribulose 1,5-bisphosphate carboxylase/oxygenase (Rubisco) which catalyses the reaction of CO_2 _with ribulose 1,5-bisphosphate (RuBP) to form two molecules of D-phosphoglyceric acid (PGA). However, it also initiates the photorespiratory nitrogen cycle by catalysing the reaction of oxygen, also with RuBP, to form one molecule each of phosphoglycolate and PGA. The precise proportion of phosphoglycolate and PGA synthesized depends on the CO_2_/O_2 _concentration ratio at the site of Rubisco inside the chloroplast and the catalytic properties of the Rubisco enzyme of the particular plant species [10-12].

Phosphoglycolate produced by the oxygenase reaction is hydrolysed in the chloroplast and the resulting glycolate is transported to the peroxisome where it is oxidised to glyoxylate by the action of glycolate oxidase, with the liberation of hydrogen peroxide that is detoxified by catalase. In the course of normal photorespiratory metabolism, the glyoxylate may be transaminated to glycine, using a range of amino acids including glutamate, serine, alanine, and asparagine. The glycine is transported to the mitochondria, where two molecules are converted to serine by a glycine decarboxylase complex and serine hydroxymethyltransferase in an oxidative process releasing equal quantities of ammonia and CO_2_. All of the ammonia released is reassimilated, probably in the chloroplast, through the combined action of glutamine synthetase (GS) and ferredoxin-dependent glutamate synthase. However the majority of the CO_2 _liberated in the mitochondria escapes to the atmosphere and is not reassimilated in C_3 _plants. Serine is transported to the peroxisome, where the amino group is transaminated to form glycine, and the other product, hydroxypyruvate, is converted to glycerate by hydroxypyruvate reductase. Finally glycerate is transported back to the chloroplast where it is recycled to PGA. The full cycle is shown in Figure 1 and the individual enzymes involved have been reviewed recently [4,5,13].

Early confirmation of the route and importance of the photorespiratory nitrogen cycle was obtained following the brilliant idea of Somerville and Ogren [14,15] that mutants deficient in specific enzymes would be able to grow normally in elevated CO_2 _when the oxygenase reaction of Rubisco was greatly decreased. However, when exposed to ambient air, the mutants would exhibit low rates of photosynthetic CO_2 _assimilation, slow growth and probably a range of stress symptoms, including necrotic lesions on the leaves. This technique led to the isolation of a range of mutants of both *Arabidopsis thaliana *and barley [14-17]. Interestingly, plants deficient in some enzymes were not isolated initially using the basic mutant screening procedure and concerns were raised as to their roles in photorespiratory metabolism. However, this was resolved later by the use of antisense and knock out techniques to specifically reduce enzyme activity, e.g. glycerate kinase [18], glutamate: glyoxylate aminotransferase [19], and glycolate oxidase [20]. Subsequent interesting findings have included a pathway via which hydroxypyruvate may also be metabolized in the cytoplasm [21], an important role of 10-formyl tetrahydrofolate deformylases in the conversion of glycine to serine [22] and an interaction between chloroplastic ferredoxin-dependent glutamate synthase and serine hydroxymethyltransferase [23] in the mitochondria. There is also evidence now that the enzymes in the cycle may be involved in metabolic processes other than just photorespiration [24,25].

Because of the loss of CO_2 _and the high energy costs of the photorespiratory N cycle, there has been considerable interest in attempts to decrease the flux through the pathway in C_3 _plants. The identification of new forms of Rubisco with an increase in the specificity for CO_2 _in relation to O_2 _has long been a target for improving C_3 _crop plants. Other targets include increasing the amount and activity of Rubisco through engineering changes in the regulatory processes of the enzyme [26-29]. C_4 _plants have evolved mechanisms that concentrate CO_2 _at the site of the Rubisco enzyme using additional enzymes, including phosho*enol*pyruvate (PEP) carboxylase and, in many cases, different cell types [6,30,31]. A number of attempts have been made to increase the activity of the C_4 _pathway enzymes in C_3 _plants, particularly in potato and rice [32,33].

A completely different proposal was put forward by Kebeish et al. [34], in which they argued that a bypass of the photorespiratory nitrogen cycle could be constructed, which would allow the metabolism of phosphoglycolate to PGA without the wasteful release of CO_2 _and ammonia. Using five genes encoding three bacterial enzymes, glycolate dehydrogenase, glyoxylate carboligase and tartronic semialdehyde reductase, they constructed a pathway inside the chloroplasts that allowed the conversion of glycolate to glycerate. The transgenic plants grew faster, produced more shoot and root biomass and contained more soluble sugars. The data suggested that although CO_2 _release was still involved, it was inside the chloroplast at the site of Rubisco activity, and that a high proportion of CO_2 _was re-assimilated [13,34].

Here we describe the phenotype, and the changes, especially in amino acids, in transgenic tobacco plants in which a pathway, similar to that which occurs in cyanobacteria, [35] has been introduced in an attempt to bypass the photorespiratory nitrogen cycle. The rationale is similar to, but distinctly different from, that used by Kebeish et al. [34]. The new pathway was engineered with two enzymes: glyoxylate carboligase (gcl; EC 4.1.1.47), which converts glyoxylate to tartronic semialdehyde and CO_2 _[36], and hydroxypyruvate isomerase (hyi; EC 5.3.1.22), which converts tartronic semialdehyde to hydroxypyruvate [37], a pathway known to operate in *E.coli *in the metabolism of glyoxylate. The enzymes were targeted to the peroxisome to make use of the glyoxylate formed by glycolate oxidase, as shown in red in Figure 1.

## Results

### Generation of transgenic tobacco plants

**Figure 2:**
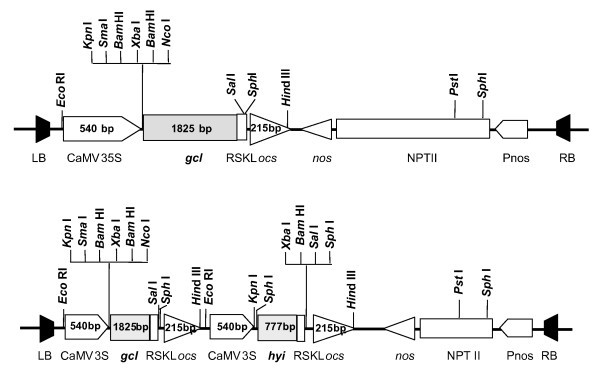
**Schematic representations of the *gcl *and *gcl-hyi *cassettes for transformation of tobacco**. **(A)**.Cassette pBIN19*gcl*. LB: left border; RB: right border; CaMV 35S: CaMV promoter; *nos*: nopaline synthase terminator; *ocs *octopine synthase terminator; *gcl*: gene encoding *E. coli *glyoxylate carboligase; *Pnos: *nopaline synthase promoter; *NPTII*: neophosphotransferase gene for kanamycin resistance. The arrowheads indicate the orientation of the promoter and terminator sequences. **(B)**. Cassette pBIN19*gcl-hyi*. LB: left border; RB: right border; CaMV 35S: CaMV promoter; *nos*: nopaline synthase terminator; *ocs *octopine synthase terminator; *gcl*: gene encoding *E. coli *glyoxylate carboligase; *hyi *gene encoding *E.coli *hydroxypyruvate isomerase; *Pnos: *nopaline synthase promoter; *NPTII*: neophosphotransferase gene for kanamycin resistance. The arrowheads indicate the orientation of the promoter and terminator sequences.

T_0 _transgenic plants were derived from tissue cultures grown from leaf disks infected with *Agrobacterium tumefasciens *containing plasmids harbouring the genes *gcl *or *gcl *and *hyi *(Figure 2) or with a similar plasmid (empty plasmid) without the genes. Positive transgenic plants were identified initially by PCR using primers designed to amplify specific segments of the *gcl *and *hy*i DNA. The phenotype, common to all individuals containing *gcl*, either alone or together with *hyi*, had chlorotic patches on the leaves when grown in ambient air under strong light conditions, but grew almost normally in air at low light intensity or in air enriched with CO_2_. Plants transformed with the empty plasmid showed no chlorotic phenotype and were used as controls in the initial selection of transgenic lines. Subsequently plants grown from seed of the original tobacco line, *Nicotiana tabacum *cv Petit Havana, were designated wild type (wt). Each original T_0 _plant was vegetatively propagated by taking stem cuttings and growing them in soil in a glasshouse, where they were heavily shaded from excess light. Six lines containing *gcl *alone and five lines containing both *gcl *and *hyi *were selected. These T_0 _plants were self pollinated and set seed. T_1 _generations were grown from this seed and homozygous individuals were selected on the basis that when self pollinated the seed produced 100% of progeny (T_2_) with the distinctive phenotype. Seeds from five such T_1 _plants, were used to produce the T_2 _plants employed in the investigation on the effects on amino acid metabolism.

### The phenotype of *gcl *transgenic lines

**Figure 3:**
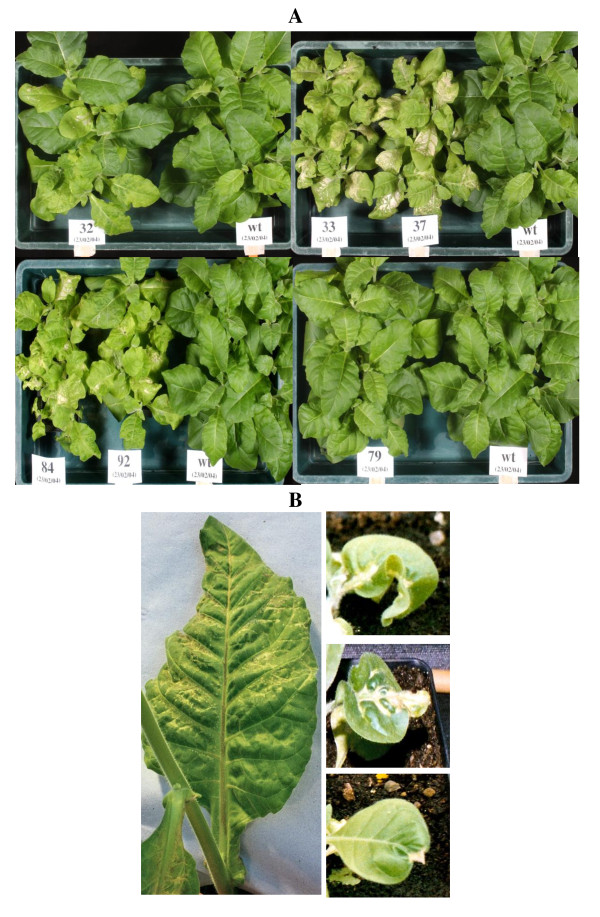
**The effect of the transformation with the *E.coli *gene *gcl *(lines 32, 33 and 37) or the *E.coli *genes *gcl *and *hyi *(lines 79, 84 and 92) on tobacco leaf phenotype**. **(A)**. The T_2 _and wild type plants were grown for 35 days in CO_2_-enriched cabinets (3000 μmol mol^-1^) then 12 days in ambient CO_2 _(350 μmol mol^-1^). **(B)**. Phenotype of line 37 transformed with *E.coli *gene *gcl*. On the left, a fully expanded leaf from an adult T_1 _plant grown for 45 days in 3000 μmol.mol^-1 ^CO_2 _and then in ambient CO_2 _in a glasshouse for 10 days. On the right are three leaves from young plants grown for 12 days at 3000 μmol.mol^-1 ^CO2 and then 7 days in ambient CO_2_.

The phenotype, common to all transgenic individuals containing either *gcl *alone or together with *hyi*, was characterised by the presence of chlorosis of the leaves following growth in ambient air. Figure 3A shows the characteristic lesions on leaves of young T_2 _transgenic plants compared to the wild type (wt), after the plants had been grown in high CO_2 _and then transferred to ambient air for 12 days. The phenotype was exhibited far less strongly in lines 32 and 79. Figure 3B shows leaves from T_1 _plants of line 37 grown at high CO_2 _and then exposed to ambient air. The mature leaf on the left of Figure 3B shows chlorotic patches near to the lateral veins and characteristic necrosis along the upper surface of the mid-rib. Leaves from young seedlings shown on the right of Figure 3B were from plants grown in elevated CO_2 _and exposed to ambient air for 7 days. Chlorotic areas were produced close to the veins with a characteristic deformation of the leaf tip and cessation of growth. Downward curvature of the leaf margins was also a characteristic of this phenotype. The lines studied all showed similar changes on exposure to air containing ambient concentrations of CO_2 _although to varying extents. Also, the phenotypes described above showed more quickly at higher light intensities.

### Expression of transferred genes in transgenic tobacco plants

**Figure 4:**
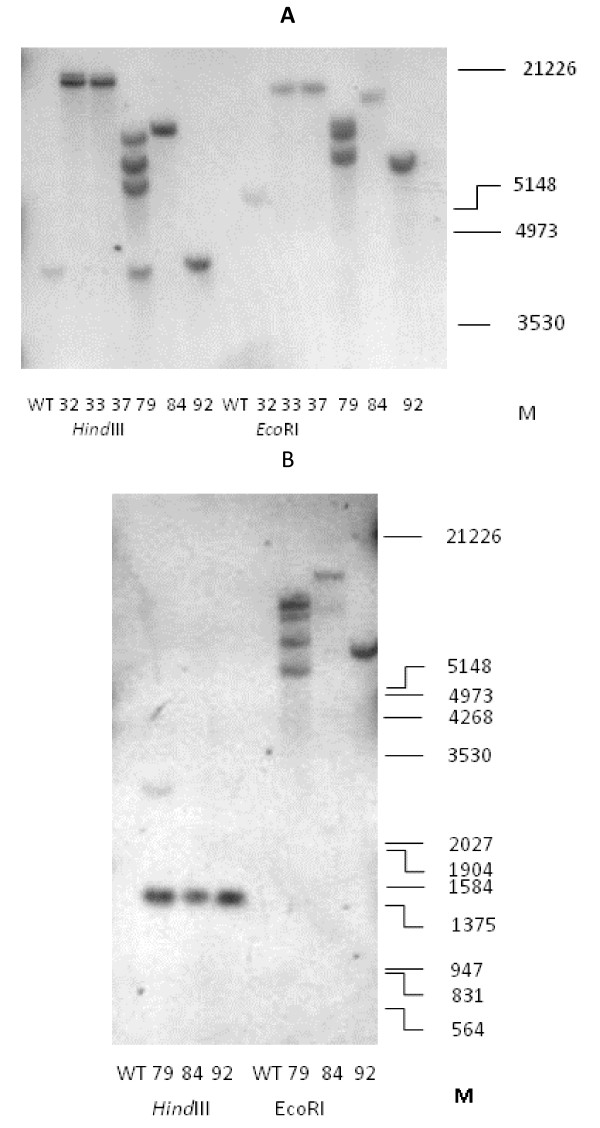
**Evidence for integration of foreign genes in transgenic tobacco plants**. **(A)**. Southern blot detection of the *E.coli gcl *gene in T_2 _transgenic lines. Genomic DNA from T_2 _plants was cut with either *Hin*dIII or *Eco*RI and probed with a *gcl *digoxygenin-labeled probe. Approximate sizes are indicated in bp. **(B)**. Southern blot detection of the *E.coli hyi *gene in T_2 _transgenic lines. Genomic DNA from T_2 _plants was cut with *Hin*dIII and *Eco*RI and probed with a *hyi *digoxygenin-labeled probe. Approximate sizes are indicated in bp.

Figure 4A shows the detection of *gcl *in six transgenic lines by Southern blotting. It was concluded that lines 32, 33, 37, 84 and 92 contained a single copy of *gcl *but that line 79 contained four copies of the gene.

Figure 4B shows the detection of *hyi *in lines 79, 84 and 92 by Southern blotting. Restriction sites close to either side of the *hyi *gene (see Figure 2B) caused digestion by *Hind*III to result in a product of similar size from each line. The results with *Eco*RI digestion, where only one restriction site within the plasmid affected *hyi*, is consistent with second sites that were more distant in the DNA bordering the inserted plasmid. As with *gcl *, the result suggests that line 79 contains four copies of *hyi*.

**Figure 5:**
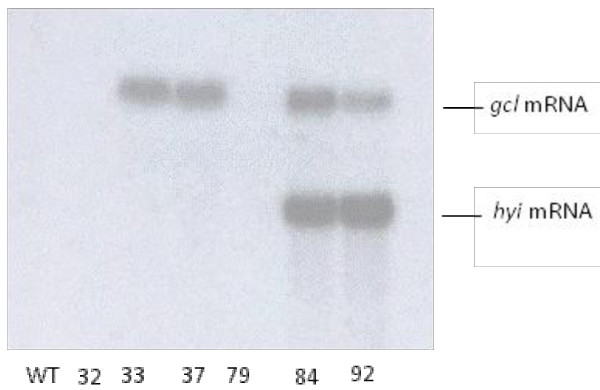
**Northern blot analysis of the expression of *E.coli gcl *and *hyi *mRNA in T_2 _transgenic lines**. The *gcl *and *hyi *mRNA was detected by probing with the ^32^P-labelled *gcl *and *hyi *genes respectively. If the membrane was incubated for 3 days a faint band corresponding to the *gcl *mRNA was also detected for line gcl 32 but not for line 79.

Figure 5 shows the result of Northern blotting for transcription of the genes in the six transgenic lines and the wt. Transcription of *gcl *was clearly detected in lines 33, 37, 84 and 92 and very slightly in 32; however, transcription of *gcl *was not detected either in line 79 or in the wt. Transcription of the *hyi *gene was again clearly detected in lines 84 and 92 but not in line 79, the gcl lines or the wt.

**Figure 6:**
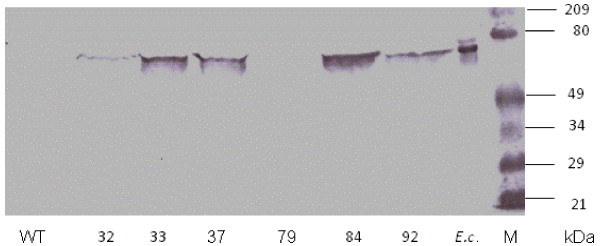
**Western blot showing expression of the *E.coli *gcl protein in T_2 _transgenic lines**. The position and sizes in kDa of the pre-stained protein standard is indicated as M. *E.co *shows the position of gcl expressed in *E. coli*.

Western blotting (Figure 6) using antisera raised against gcl expressed in *E. coli*, showed that there was clear expression of a protein of approximate molecular mass of 66 kDa in lines 33, 37, 84 and 92, weak expression in line 32 and none in the wt and 79. Using antisera raised against a KLH peptide (see Materials and Methods), no evidence of expression of a hyi protein in any of the transgenic plants was obtained by western blotting, although a 29 kDa protein was detected in extracts of *E.coli *cells expressing hyi (result not shown).

### Metabolism of glycolate

**Figure 7:**
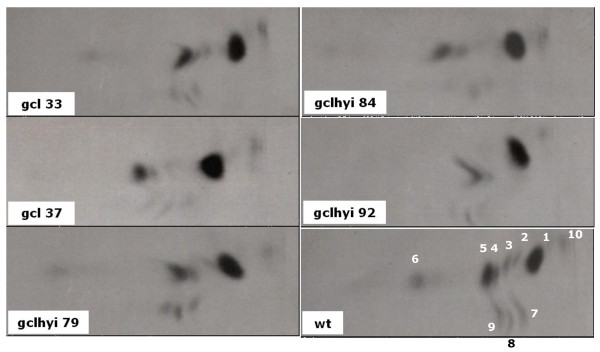
**Autoradiograms of thin layer cellulose chromatograms showing the products of metabolism of [^14^C]glycollate in leaf strips from wild type and T_2 _transgenic plants**. The plants were grown in high CO_2 _for 35 days and were then transferred to ambient air for 3 days. Radioactivity was found in glycollate (1), ala (2), glycerate (3), gly (4), ser (5), sucrose (6), malate (7), glu (8) and asp (9) in the wild-type. There was a radioactive impurity (10) present in the sample of [^14^C]glycolate, probably monochloroacetate, that appeared in all of the chromatograms. Chromatograms were developed using n-propanol/0.880 sg ammonia solution/H_2_O (6:3:1) in the first dimension (vertical in the Figure) and twice with n-propylacetate/90% formic acid/H_2_O (11:5:3) in the second dimension.

Because of the development of lesions on leaves of the transgenic plants in the ambient atmosphere, it was necessary to grow the plants in high CO_2 _and make measurements of metabolism after a relatively short time of exposure to the ambient air. In the main experiments, we studied the metabolism 3 days after transfer from the high CO_2_. Strips of leaves were supplied with [^14^C]glycolate in the transpiration stream in ambient air, in the light (Figure 7). The main products detected were serine and glycine. Although the ^14^C-labelling in glycine and serine appeared to be somewhat less in the leaf strips of the transgenic lines compared to the wt, it was concluded that a flux into the photorespiratory nitrogen cycle still existed in the transgenic plants.

### Amino acid and sugar analysis of transgenic plants

As indicated above, the chlorotic phenotype appeared only after several days of exposure to ambient air following growth of the transgenic lines in elevated CO_2_. Initial attempts to detect reproducible, early changes in metabolism following transfer to ambient air were not successful and therefore a different type of experiment was devised. Newly germinated seedlings were grown in elevated CO_2 _(3000 μmol mol^-1^) in a controlled environment (CE) cabinet for 30 days, whereupon half of the plants were transferred to a CE cabinet containing ambient CO_2 _(350 μmol mol^-1^). After 3 days, three independent samples each comprising 9 shoots of each line were taken from both the ambient air and the elevated CO_2 _CE cabinets. The samples of shoots were immediately powdered in liquid nitrogen and freeze-dried.

**Figure 8:**
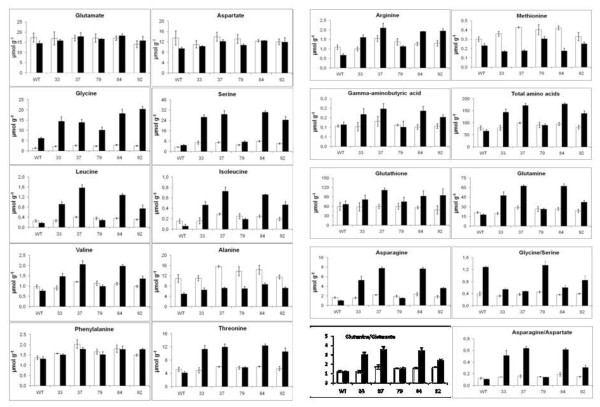
**The effect of transfer from elevated CO_2 _to ambient CO_2 _on the content of amino acids in tobacco shoots**. Plants were grown for 30 days in elevated CO_2 _(3000 μmol mol^-1^) and half were transferred to ambient CO_2 _(350 μmol mol^-1^) for 3 days. The results, μmol g^-1 ^dry mass, are means for three replicates ± SEM, open bars ■ for plants at elevated CO_2_, solid bars ■ for plants after 3 days in ambient CO_2_. Statistical evaluation in: Additional Files, Additional File 1 Tables S1, S2a, S2b, S2c.

Figure 8 shows the amino acid content of the shoot sub-samples of the freeze-dried powders described above, analysed by HPLC. The values presented are the means of measurements on the three biological replicates. Statistical analysis (Supplementary Material, Additional File 1) showed the following significant results (p < 0.05). The leaves of all transgenic lines contained significantly larger quantities of soluble glycine and serine than the wt under both high and ambient CO_2 _conditions. Also, for all the transgenic lines and the wt, the quantities of glycine and serine were significantly less in the plants under high CO_2 _conditions. Amongst the other amino acids, all five of the transgenic lines contained significantly larger quantities of soluble leucine, isoleucine, valine, alanine, phenylalanine, threonine, arginine, glutamine, asparagine and total amino acids than the wt under ambient CO_2 _conditions; but also, for these amino acids, two transgenic lines had significantly greater quantities than the wt under high CO_2 _conditions, except that no significant difference of threonine was observed compared to the wt. There was also overall significantly greater tyrosine, γ-aminobutyric acid and glutathione observed in transgenic lines under low CO_2 _conditions and aspartate under high CO_2 _conditions, with line 37 showing significantly greater aspartate compared to the wt.

The amounts of total glutathione, [reduced glutathione (GSH) plus oxidised glutathione (GSSG)] were increased in the shoot samples of the transgenic lines exposed to ambient air but not in those grown continuously in elevated CO_2_. Whereas the amounts of GSH + GSSG were increased five to six fold in the leaves of lines 32 and 92 in T_0 _plants growing in a glasshouse in ambient air, when compared to the wt (data not shown).

**Figure 9:**
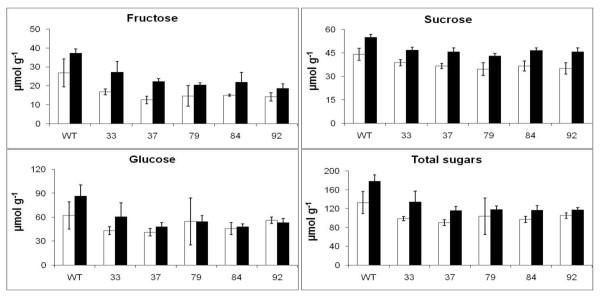
**The effect of transfer from elevated CO_2 _to ambient CO_2 _on the content of sugars in tobacco shoots**. Plants were grown for 30 days in elevated CO_2 _(3000 μmol mol^-1^) and half were transferred to ambient CO_2 _(350 μmol mol^-1^) for 3 days. The results, μmol g^-1 ^dry mass, are means for three replicates ± SEM, open bars ■ for plants at elevated CO_2_, solid bars ■ for plants after 3 days in ambient CO_2_. Statistical evaluation in: Additional Files, Additional File 1 Tables S3 and S4.

Figure 9 shows the quantities of sugars in sub-samples of the same freeze-dried powders of shoots from transgenic lines used to produce the amino acid results in Figure 8. Glucose, fructose and sucrose were significantly lower (p < 0.05) in all the transgenic lines than in the wt in ambient CO_2_. There was a greater accumulation of total sugars in the low compared to high CO_2 _conditions (Additional Files, Additional File 1).

## Discussion

The transgenic lines exhibited chlorotic lesions close to the veins when exposed to ambient air. In four of the six transgenic lines chosen for further study (lines 33 and 37 transformed with the *gcl *gene only, and 84 and 92 transformed with the combined *gcl-hyi *gene), the lesions were severe (Figure 3). The chlorotic phenotype was less visible in the leaves of line 32 and hardly at all in 79. Transgenic line 32 was not included in the final stages of the investigation as the gcl protein was only weakly expressed, possibly because the plants may not all have been homozygous. All transgenic lines appeared to grow normally in elevated CO_2_, except that germination of the seed was slower. The data indicated that the *E.coli gcl *gene had been transferred as one copy into lines 33, 37, 84 and 92 (Figure 4A) and that the gene could be transcribed and translated to form a polypeptide of the correct molecular mass (Figures 5 and 6). Similarly, the data indicated that lines 84 and 92 had been transformed with one copy of the *E.coli hyi *gene and that the gene was transcribed to form mRNA. However, antibody raised against a short hydrophilic sequence of the hyi protein that recognised the native *E. coli *protein, did not cross react with any protein in extracts of the transgenic leaf. This would suggest that either the mRNA was not translated or that the hyi polypeptide was immediately subjected to proteolysis following synthesis. The Southern blots in Figure 4A and 4B indicate that four copies of the combined *E.coli gcl-hyi *gene had been transferred into line 79; however, there was no evidence of transcription of any mRNA or translation of any protein. Co-suppression and gene silencing phenomena of multiple genes that have been transferred into plants are now well established [38] and this was presumably what had occurred here.

Stunted growth and the presence of lesions on the leaves following exposure to ambient air, but the ability to grow more normally in air with an elevated concentration of CO_2_, is a characteristic of the photorespiratory mutants originally isolated in *A. thaliana *and barley and suggests that there is a metabolic defect related to the photorespiratory nitrogen cycle [14,17,39]. If a fully operative gcl/hyi pathway as shown in Figure 1 was present in any of the transgenic plants, a decrease in flux into glycine and serine would be predicted. The concentrations of the two amino acids glycine and serine, which are direct metabolites in the photorespiratory nitrogen cycle, increased to varying extents following transfer from elevated CO_2 _to ambient air (Figure 8). This increase is consistent with an increased rate of oxygenation of RuBP (*v_0_*) and flux into the photorespiratory pathway. Likewise the over three fold increase in the ratio of glycine/serine in the leaves of the wt and 79 upon transfer to ambient air is consistent with an increased *v_0 _*and photorespiratory flux [40]. The smaller effect on the ratio of glycine/serine in the transgenic lines 33, 37, 84 and 92 following the transfer to photorespiratory conditions, is consistent with some diversion of glyoxylate away from glycine into the synthesis of tartronic semialdehyde. In addition, Novitskaya et al. [40] also demonstrated that the aspartate and alanine contents exhibited negative correlations with the photorespiratory flux. Figure 8 shows that the alanine content of leaves was decreased following transfer to air in both the wt and in the transgenic lines, whilst that of aspartate showed less changes. This would suggest that alanine but not aspartate is metabolized under the photorespiratory conditions of ambient air in all plants, irrespective of the introduction of the foreign *gcl *and *hyi *genes [19].

The presence of both glycine and serine in the leaves of the transgenic lines in ambient CO_2 _suggested that the phenotype was not caused by a total block of the photorespiratory nitrogen cycle. The incorporation of ^14^C from [^14^C]glycolate into glycine and serine indicated flux into the photorespiratory nitrogen cycle. It is presumed that the metabolic flux catalysed by glyoxylate carboligase through to tartronic semialdehyde, however slow it may be, directly or indirectly caused the chlorotic lesions on the leaves.

Somewhat surprisingly the necrotic lesions initially developed close to the veins, whereas, during the normal senescence of tobacco leaves, chlorophyll breakdown starts in the mesophyl in the interveinal areas [41]. There is evidence that senescence may be induced by a reduction in peroxisomal catalase activity and an increase in hydrogen peroxide [41,42], however this interaction may be complex [43]. Mutant and antisense lines deficient in catalase exhibit chlorophyll bleaching and necrosis in the interveinal areas, a phenotype that can be rescued by elevated CO_2 _or low light [44,45]. Therefore, if the necrotic lesions of the transgenic lines are directly or indirectly caused by a build up of peroxide, this must be initially generated in or near tissues associated with the veins.

Takahashi et al. [46] proposed that in mutants with lesions, in their photorespiratory nitrogen cycle there is a reduction in Calvin cycle intermediates because of a decrease of the activity of some specific enzymes [39]. The ensuing decrease in the flux through the Calvin cycle decreases the consumption of ATP and NADPH and results in an imbalance between the production of photochemical energy and its consumption in carbon assimilation. Under such conditions, electrons originating from the oxidation of water at PSII are transferred to oxygen at PSI and produce the reactive oxygen species (ROS), O_2_^- ^and H_2_O_2 _[47]. The ROS inhibit the repair of photodamaged PSII, owing to suppression of the synthesis of the D1 protein, thus causing photoinhibition [48,49] and eventually chloroplast damage. Our results suggest that there was an increase in glutathione in the plants containing gcl. Recent proposals by Foyer and Noctor (2011) have suggested that changes in glutathione may be able to transmit ROS signals within the plant [50].

The data in Figure 8 show that there is an increase in the majority of soluble amino acids, in particular the amides asparagine and glutamine, in the leaves, especially of lines 33, 37, 84 and 92, when compared to the wt, following exposure to air. However, in the leaves of line 79, where there is evidence of co-suppression, there is no major accumulation. Thus there is a correlation between expression of the gcl protein, leaf necrosis and amino acid accumulation. The question is whether the accumulation of amino acids and amides is a consequence of the chlorosis, or the cause. During the normal senescence of leaves of both tobacco and *A. thaliana*, the soluble amino acid concentration decreases with leaf age even though the protein is being hydrolysed [51,52]. The result suggests that the necrosis in the vascular area of the transgenic plants described above prevents the efficient transport of proteolysis-liberated amino acids out of the senescing leaf, as happens in detached leaves [53]. Alternatively, the increase in amino acids could be due to a general response to stress, cessation of protein synthesis or a decrease in the degradation of particular amino acids. That there was no comparable accumulation of sugars due to such a vascular dysfunction is reasonably explained by the decreased carbon assimilation due to destruction of the photosynthetic apparatus.

The accumulation of asparagine and glutamine is normally an indicator of a stress condition where protein synthesis has been inhibited and there is a plentiful supply of reduced nitrogen [53]. In contrast, a relatively constant concentration of glutamate under a wide range of external conditions is an indicator of a homeostatic mechanism [54]. Interestingly, Noctor et al. [55] provided evidence that there was a linear relationship between the minor soluble amino acids in wheat, potato and barley leaves, and total amino acids, indicating that the amino acid contents are co-ordinated across biosynthetic families.

Barley mutants unable to convert glycine to serine during photorespiration had lower NADH/NAD ratios in the mitochondria and higher ratios in the cytoplasm. There was also evidence of increased malate oxidation in glycine decarboxylase deficient lines of both barley and potato [56,57]. As shown in Figure 1, in order to balance the photorespiratory nitrogen cycle as a whole, it is necessary that the reducing equivalents from glycine oxidation in the mitochondria are transferred to the peroxisome to drive the reduction of hydroxpyruvate to glycerate. It has always been assumed that this process is carried out by a malate/oxaloacetate shuttle involving malate dehydrogenase in both the mitochondria and peroxisomes.

The role of mitochondrial malate dehydrogenase in photorespiration has been confirmed but the importance of peroxisomal malate dehydrogenase is less clear [58]. If the glyoxylate is diverted directly to hydroxypyruvate through glyoxylate carboligase then there will be a deficiency in reducing power generated through the glycine/serine conversion, which would have to be supplied by a different mechanism if hydroxypyruvate reductase is to operate. As there are both NADH- and NADPH-dependent forms of hydroxypyruvate reductase [21], it is possible that the NADH could be supplied from a reactivated tricarboxylic acid cycle in the mitochondria, or that NADPH would be available from the chloroplast [59-61], due to a decreased demand for ammonia assimilation.

It is worth considering what effects the insertion of a fully operative gcl/hyi pathway, as shown in Figure 1, would have on photosynthetic metabolism as a whole. The first point is that there would be no release of ammonia and hence no need for the photorespiratory nitrogen cycle, and no need to recycle glutamate and 2-oxoglutarate. There would therefore be a saving of ATP and reduced ferredoxin in the chloroplast from the GS and GOGAT reactions, whilst NADH would not be generated in the mitochondria, following the glycine to serine conversion. The CO_2 _liberated in the mitochondria would now be formed in the peroxisomes by the gcl reaction. However, as there is evidence that there is a specific protein (PEX10) on the single membrane of the peroxisome that allows attachment of peroxisomes to chloroplasts [62], it is possible that the CO_2 _would diffuse more readily to the site of Rubisco activity rather than if it had been generated in the mitochondria.

The initial step in the conversion of glycine to serine requires the action of a four peptide-containing glycine decarboxylase enzyme. The methylene group remaining from glycine following the removal of CO_2_, ammonia and NADH synthesis is transferred to tetrahydrofolate (THF) to form 5, 10-methylene-THF, which is then transferred to a second molecule of glycine, catalysed by serine hydroxymethyltransferase. The role of 10-formyl THF deformylase during photorespiration has recently been demonstrated [22]. One carbon metabolism using serine as a donor is important in the synthesis of a range of methylated compounds including nucleic acids, proteins and chlorophyll. [63-65]. A range of mutant lines of *A. thaliana *and barley have been isolated and characterized that have deficiencies in the double enzyme complex that is required to convert glycine to serine [66-69]. Initially it was demonstrated that such mutants were able to grow normally in elevated CO_2_, indicating the complex was only required for the photorespiratory nitrogen cycle and was not required for the synthesis of other C1-THF derivatives. Other pathways of C1 metabolism were proposed, some involving formate [64,70]. However, in an elegant series of experiments, Engel et al. [24] demonstrated that knocking out both genes encoding the P protein of glycine decarboxylase of *A. thaliana *produced a lethal mutant that was unable to grow past the seedling stage even at elevated concentrations of CO_2_. As the transgenic lines in these experiments contained high concentrations of both glycine and serine, it seems unlikely that C1-THF metabolism is a limiting factor.

## Conclusions

In conclusion, transgenic tobacco plants have been generated that produce bacterial glyoxylate carboligase but not hydroxypyruvate isomerase. Evidence presented shows that the photorespiratory nitrogen cycle was not completely by-passed. The transgenic plants exhibit a stress response when exposed to air that suggests that some glyoxylate is diverted away from glycine in a deleterious short-circuit of the photorespiratory nitrogen cycle. This diversion in metabolism gave rise to necrosis and increased concentrations of amino acids, in particular the amides glutamine and asparagine, in the leaves and a decrease in soluble sugars in the shoot.

Extension of the transformation strategy described would require that the reasons for the lack of expression of the hyi protein must be investigated. Hydroxypyruvate and tartronic semialdehyde are labile and reactive compounds that are interconvertible under alkaline conditions without the presence of the hyi enzyme. Even with the expression of active enzymes of both gcl and hyi, it may be necessary to undertake further manipulation to down-regulate the aminotransferases that catalyse the amination of glyoxylate. This should then allow a rapid transfer of the glyoxylate to hydroxypyruvate within the peroxisome and negate the requirement for photorespiratory ammonia assimilation.

## Methods

### Plant growth conditions

Tobacco (*Nicotiana tabacum *L) plants cv Petit Havana were used as the experimental wild type (wt) material. Seeds were germinated on filter paper and seedlings grown on in pots or trays containing peat based compost supplemented with Osmocote slow release fertilizer, either in a glasshouse or in controlled environment (CE) cabinets. In the glasshouse, the photosynthetically active radiation (PAR) was maintained at between 300 and 600 μmol m^-2 ^s^-1 ^in a light/dark cycle of 16 h/8 h. Conditions in the CE cabinets were set to provide the PAR at plant level of 300 μmol m^-2 ^s^-1^, a light/dark cycle of 14 h/10 h, a temperature of 25°C/20°C, and a relative humidity of 70/80%. Depending on the experiments, the CO_2 _concentration in the cabinets was controlled light/dark at either 350/350 or 3000/350 μmol mol^-1^.

### Tobacco transformation and selection of transgenic lines

The pYYC160 plasmid containing the *E.coli gcl *gene encoding glyoxylate carboligase was a gift from Dr Y.Y. Chang [36] and the pHYI1 plasmid containing the *E. coli hyi *gene encoding hydroypyruvate isomersase was a gift from Dr M.Ashiuchi [37]. Both genes were amplified by PCR with primers including a 3' extension sequence encoding the peroxisome targeting sequence, RSKL. The PCR products were cloned into pUC18 to create plasmids pGCL3 and pUCP*hyi*T and sequenced. The plasmid pBIN19AR [71] used to make the two vectors for the transformation of tobacco was a gift from Professor M. Stitt, and includes the CaMV 35S promoter and octopine synthase terminator sequences.

The plasmid pBIN19*gcl *(Figure 2), was constructed from pBIN19AR and pGCL3, encoding a gcl protein with a 3' extension sequence encoding the peroxisome targeting sequence RSKL. The *hyi *gene with a 3' extension sequence encoding the peroxisome targeting sequence was incorporated into pBIN19*gcl *to form the pBIN19*gcl-hyi *plasmid (Figure 2), so that both genes could be used for transformation of tobacco at the same time.

The plasmids were introduced into the wt tobacco by *Agrobacterium tumefaciens*-mediated transformation of leaf explants. Plantlets were regenerated by organ-tissue culture and adapted *ex-vitro *in a controlled environment. The T_0 _plants were screened by PCR with construct-specific primers to identify those containing the *gcl *or *gcl *and *hyi *genes. A phenotype for plants carrying the genes was recognised by chlorosis of leaves growing in normal light and in ambient CO_2_. The T_0 _lines retained included line 4, transformed with an empty plasmid, lines *gcl *6, *gcl *15, *gcl *32, *gcl *33, *gcl *37 and *gcl *38, transformed with pBIN19*gcl *and the lines *gcl-hyi *79, *gcl-hyi *83, *gcl-hyi *84, *gcl-hyi *89, *gcl-hyi *92, transformed with pBIN19*gcl-hyi*. Plants of these lines were propagated from stem cuttings in low light in a glasshouse. Lines *gcl *32, *gcl *33, *gcl *37, *gcl-hyi *79, *gcl-hyi *84 and *gcl-hyi *92 were retained for further study. For the sake of simplicity, the transgenic lines have only been referred to by their numbers in the text. T_1 _seeds produced following self-pollination of the selected T_0 _lines were germinated for 12 days, and seedlings were subsequently grown in 48 cm^3 ^pots in CO_2_-enriched (3000 μmol mol^-1^) CE cabinets. After 15 days, the seedlings were transferred to a glasshouse for 7 days in ambient CO_2_. Seedlings showing chlorosis were re-potted into 854 cm^3 ^pots and transferred back to CO_2_-enriched cabinets to flower and to produce seeds following self-pollination. Samples of T_2 _seeds from the selected T_1 _plants exhibiting the phenotype were germinated and the seedlings established in CO_2_-enriched cabinets and then subjected to growth in ambient CO_2_, in a glasshouse. Individual T_1 _plants that produced 100% of T_2 _seedlings showing chlorosis were selected. Seeds from these T_1 _plants were used to produce the T_2 _plants for the experimental studies.

### Extraction and characterisation of DNA and RNA

Genomic DNA was isolated from approximately 1 g of leaf tissue using a cetyltrimethylammonium bromide (CTAB) procedure [72]. Samples of DNA, including PCR products and probes were separated and characterised by electrophoresis in agarose gels containing ethidium bromide, and visualised in UV light. RNA was isolated, using a Trizol^® ^(Invitrogen) procedure, from 100 mg samples of leaf tissue ground to a powder in liquid nitrogen. RNA was separated and characterised by electrophoresis in denaturing agarose gels.

### Preparation of *E.coli *extracts containing gcl and hyi proteins

The *gcl *gene was transferred from pGCL3 to the Qiagen expression plasmid pQE31 to create an *E.coli *expression plasmid pQE31*gcl*. The *hyi *gene was transferred from pUCP*hyi*T to the Qiagen expression plasmid pQE32, to create *E.coli *expression plasmid pQE32*hyi*. 5 mL cultures of colonies transformed with these plasmids were grown for 3 hours in Luria-Bertani (LB) broth containing 5 μL of ampicillin (100 mg mL^-1^) and 3 μL kanamycin (50 mg mL^-1^), protein expression was induced by the addition of Isopropyl β-D-thiogalactosylpyranoside (IPTG) to a final concentration of 0.5 mM and the culture was grown overnight. Next morning, cells from 200 μL aliquots collected by centrifugation, were resuspended in 2× SDS loading buffer (0.06 M Tris-HCl, pH 6.8, 10% v/v glycerol, 2% v/v SDS, 0.1% v/v saturated bromophenol blue), and heated at 95°C for 3 min and analysed by denaturing polyacrylamide gel electrophoresis.

### Southern blot analysis

Southern blots were prepared [73] using kits supplied by Roche Applied Science and following the instructions supplied for luminescent detection of DIG (digoxygenin) labelled DNA. Probes were prepared using DNA cut from plasmids pQE31*gcl *and pQE32*hyi *with *Eco*R1 into which DIG-dUTP label was incorporated using the PCR DIG Probe Synthesis Kit (Roche). The *gcl *probe consisted of a PCR-product of 554 bp starting at position 381 of the *gcl *gene to position 924 [36]. The *hyi *probe consisted of a PCR-product of 441 bp starting at position 97 of the *gcl *gene to position 537 [37]. Approximately 15 μg of genomic DNA was denatured at 65°C for 10 min and digested with 60 U *Eco*RI or *Hin*dIII and the products separated by electrophoresis in a 0.8% agarose gel containing ethidium bromide alongside a DNA molecular weight marker III (Roche). After denaturing, the DNA was transferred to a positively charged nylon membrane (Roche) and cross-linked to the membrane by exposure to UV light. After hybridisation with probes, the membrane was washed and blocked following the manufacturer's instructions before treating with a solution of anti-DIG alkaline phosphatase Fab Fragments (Roche). After washing away the excess phosphatase, the membrane was sealed in a plastic bag containing disodium 3-(4-meth-oxyspiro (1,2-dioxetane-3-2'-(5'-chloro)tricyclodecan)4-yl)phenylphosphate (CSPD) and exposed to Kodak^® ^X-Omat film XAR.

The DIG-labelled *gcl *probe was removed by rinsing the membrane thoroughly in double distilled water and then twice in probe removal solution (0.2 M NaOH, 0.1% w/v SDS) at 37°C for 15 min. After rinsing in 0.3 M NaCl, 0.03 M tri-sodium citrate at room temperature, the membrane was blocked following the manufacturer's instructions and hybridised with the *hyi *probe.

### Northern blot analysis

The restriction enzymes *Kpn*I and *Sph*I (Fermentas) were used to excise *hyi *DNA from pUChyiT and *Bam*HI was used to recover *gcl *DNA from pGCL3. Both samples of DNA were purified by electrophoresis and labelled with ^32^P using a Prime-a-Gene^® ^labelling system kit (Promega) according to the manufacturer's instructions. RNA purified from the leaf tissue was separated on denaturing agarose gels and transferred to a Hybond™ NX, nylon membrane and cross linked by exposure to UV light. The RNA was denatured and exposed to the DNA probes at 65°C overnight. After washing to remove background radioactivity, membranes were exposed to Kodak^® ^Biomax MS film.

### Western blot analysis

Fresh leaf material (4 × 3.1416 cm^2 ^discs) was ground to a fine powder in liquid nitrogen and then in 1 mL of 2× SDS loading buffer. The homogenate was heated to 95°C for 3 min and then centrifuged at 16,000 × g at 4°C for 15 min. The supernatant was removed to a clean tube, vortexed and 35 μL samples (approx 60 μg protein) were separated by denaturing polyacrylamide gel electrophoresis. The proteins were electro-transferred from the polyacrylamide gels to a 0.45 μm nitrocellulose membrane (Bio-Rad) at 100 V. After blocking with 5% (w/v) non-fat milk, the membrane was incubated with a solution of the primary antibody, with anti rabbit IgG horseradish conjugate and finally with 20 μM 4-chloronaphthol.

The primary antibody to gcl was raised against protein purified from *E.coli *cells. *E. coli *M15[pREP4] cells were transformed with plasmid pQE30*gcl*, and, following induction with 1 mM IPTG, expressed gcl. The protein was purified for antibody preparation by the method suggested by the manufacturer [74]. Lysozyme was needed to release the protein from the bacterial cells; the protein was bound to a Ni-NTA agarose column and was recovered in the fraction eluting in buffer containing 229 mM imidazole. When the eluate was dialysed against phosphate buffered saline (PBS), the protein precipitated. However, precipitated protein was dissolved by raising the pH to 9.5 and the solution was freeze dried. This protein was used to generate rabbit antiserum.

For hyi, the primary antibody used was raised to a sequence DNPHRGEPGTGEINY located towards the carboxy terminus of the *E.coli *hyi protein in rabbit, in conjunction with keyhole limpet haemocyanin (KLH).

### The metabolism of [^14^C]glycolate

Strips of the tobacco leaves (75 × 5 cm) were cut under water to include a lateral vein. The strips were supported vertically with the cut bases in a 10 mM solution of [^14^C]glycolate (370 kBq of ^14^C per μmol), and illuminated with white light with a photosynthetic photon flux density (PPFD) 800 μmol m^-2 ^s^-1^. After 50 min the strips were dropped into 2 ml of boiling 50% ethanol and boiled for 2 min. The extract together with one further extract made with 2 ml of boiling ethanol was dried down and the residue was dissolved in a small volume of 50% ethanol and applied to a Whatman K2 cellulose thin layer (250 μm) chromatography plate. The products were separated by development in 2-dimensions [44].

### Analysis of amino acids and glutathione

Amino acids and glutathione were analysed by HPLC after reaction with o-phthalaldehyde and 2-mercaptoethanol using the methods of Noctor and Foyer [75] and Novitskaya *et al*. [40] with minor modifications. Sub-samples of freeze-dried powders of shoots were extracted in 0.1 M HCl, following which α-aminobutyric acid was added as an internal standard to the clarified extract. The extracts were analysed using a Waters HPLC system consisting of a Symetry^® ^C18 3.5 μm-4.6 × 150 mm column equipped with a guard column, a W 2695 separation module, and a W474 scanning fluorescence detector all controlled by a Millenium Chromatography Manager workstation running the Millenium^32 ^software version 4.0 (Waters, Elstree, Hertfordshire, UK). The eluents used were A, 80% v/v 50 mM sodium acetate, pH 5.9, 19% v/v methanol, 1% v/v tetrahydrofuran; and B, 80% v/v methanol, 20% v/v 50 mM sodium acetate, pH 5.9; the flow rate was 0.8 mL min^-1^. The eluent at 0, 1, 6, 11, 16, 20, 32, 40, 41 and 46 minutes contained 100, 100, 90, 90, 55, 55, 0, 0, 100 and 100% of A respectively.

### Analysis of sugars

Sub-samples of freeze-dried powders of shoots were sequentially extracted with 1.5 mL 90% (v/v) ethanol at 60°C for 3 min, 1 mL 50% ethanol at 60°C for 3 min and a further 1 mL 50% ethanol at room temperature. After each extraction, the tube was centrifuged at 16,000 × g for 5 min at room temperature. The combined supernatants were made up to 5 mL with 50% ethanol. Two hundred μL was evaporated to dryness using a Speed Vac concentrator and the residue was re-dissolved in 1.0 mL H_2_O. The solution was vigorously vortexed and clarified by centrifugation for 5 min at 16,000 × g at 20°C. After filtering through a syringe filter into an autosampler vial, the sugars were analysed using a Dionex (Sunnyvale, CA, USA) DX-500 ion chromatography system consisting of a CarboPac™ PA1 4 × 250 mm column, a CarboPac PA1 (4 × 50 mm) guard column, a quaternary gradient pump (GP40), an electrochemical detector (ED40) with a gold working electrode, a pH-Ag/AgCl reference electrode, a Polyether ether ketone (PEEK) rotary injection valve, and an AS3500 autosampler. A personal computer equipped with the Dionex PeakNet™ 4.31 Windows based software automated the system operation. The sugars were separated using a linear gradient of NaOH from 0.04 to 0.5 M in 20 min.

### Statistical evaluation of measurements of amino acids and sugars

Analysis of Variance (ANOVA) was used to assess the statistical significance of the effects of line, CO_2 _and the interaction between these two factors for each of the amino acids, sugars and the ratio of some pairs of amino acids of interest (Additional Files, Additional File 1). A natural log transformation (to base *e*) was used for all data to account for heterogeneity of variance across the treatment combinations in application of ANOVA. For Lys and Tyr, a small adjustment of 0.005 was used so that zero observations of these amino acids could be included in the analysis under the log transformation. Following ANOVA, least significant difference (LSD) values at the 5% (p = 0.05) level of significance were used to compare means of particular interest, specifically those of the lines to the wt control.

## Competing interests

The authors declare that they have no competing interests.

## Authors' contributions

JdeFCC made measurements of metabolites, recorded the phenotype under various conditions, determined the presence, transcription and translation of the genes and generated the first draft of the manuscript. PJM created the constructs for transformation of tobacco plants, took the plants through stages of selection and crossing to select homozygous plants, and advised on all molecular biology techniques. AJK advised and helped with analytical methods, general laboratory techniques and preparation of the manuscript for publication. PJL together with MAJP conceived the project and were responsible for the overall supervision of the research. PJL made a major contribution to the preparation of the manuscript for publication. SJP made the statistical analysis of the measurements of amino acids and sugars. All of the Authors made contributions to the preparation of the manuscript and have read and approved the final version.

## Acknowledgements and Funding

The Authors thank Dr YY Chang, Department of Microbiology and Biochemistry, University of Illinois at Urbana-Champaign, Urbana, Illinois 61801, USA, for plasmid pYYC160 containing the *E.coli gcl *gene, Dr M Ashiuchi, Department of Bioresources Science, Faculy of Agriculture, Kochi University, Nankoku, Kochi 783-8502, Japan, for plasmid pHYI1 conaining the *E.coli hyi *gene, and Professor M Stitt, MaxPlanck Institute of molecular Plant Physiology, Am Muehlenberg 1, Golm 14476, Germany, for the plasmid pBIN1AR. Josirley F.C. Carvalho was supported by a fellowship from CNPq (National Council for Scientific and Technological Development) Brazil. Rothamsted Research is an institute of the Biotechnology and Biological Sciences Research Council of the UK.

## Supplementary Material

Additional File 1 **The statistical evaluation of the measurements of amino acids and sugars**. This file includes Tables S1, S2a, S2b, S2c, S3 and S4 together with explanatory text. Click here for file
