# Supplementary material for: An engineered pathway for glyoxylate metabolism in tobacco plants aimed to avoid the release of ammonia in photorespiration
Source: BMC Biotechnol. 2011 Nov 21;11:111. doi: 10.1186/1472-6750-11-111 (PMC3252329; doi:10.1186/1472-6750-11-111)
Supplement: Additional File 1 — The statistical evaluation of the measurements of amino acids and sugars. This file includes Tables S1, S2a, S2b, S2c, S3 and S4 together with explanatory text. [file 1472-6750-11-111-S1.DOC]

**Additional Files**

Additional File 1

The statistical evaluation of the measurements of amino acids and sugars with Tables S1, S2a, S2b, S2c, S3 and S4.

**Amino Acids**

The table below shows the statistical significance (p-values) of the main effects of line (WT, 33, 37, 79, 84 and 92) and treatment (low CO_2_ (air) and high CO_2_ (3000 μmol mol^-1^) and the interaction between these two factors for each amino acid.

**Table S1. Significance (F-statistics, p-values) of main effects of line, treatment and the interaction between these factors. F-statistics are on 5 and 24 degrees of freedom (df) for the main effect of line and the interaction, and on 1 and 24 df for the main effect of Treatment. P-values indicating corresponding means tables to investigate are highlighted in bold.**

| **Amino Acid** | **Line** | **Treatment** | **Line.Treatment** |
| --- | --- | --- | --- |
| Log(Glu) | 2.13, 0.096 | 0.03, 0.873 | 1.37, 0.272 |
| Log(Asp) | 2.72, **0.044** | 12.07, **0.002** | 2.26, 0.081 |
| Log(Gly) | 50.40, < 0.001 | 1927.77, <0.001 | 6.18, **< 0.001** |
| Log(Ser) | 198.27, < 0.001 | 1064.17, < 0.001 | 44.98, **< 0.001** |
| Log(Leu) | 89.83, < 0.001 | 242.56, < 0.001 | 56.54, **< 0.001** |
| Log(Iso) | 25.83, < 0.001 | 19.04, < 0.001 | 14.53, **< 0.001** |
| Log(Val) | 45.84, < 0.001 | 88.78, < 0.001 | 29.91, **< 0.001** |
| Log(Ala) | 15.75, < 0.001 | 383.01, < 0.001 | 3.38, **0.019** |
| Log(Phe) | 17.62, < 0.001 | 0.96, 0.338 | 3.20, **0.024** |
| Log(Thr) | 39.46, < 0.001 | 197.73, < 0.001 | 32.18, **< 0.001** |
| Log(Arg) | 46.05, < 0.001 | 23.57, < 0.001 | 25.92, **< 0.001** |
| Log(Met) | 8.87, < 0.001 | 288.77, < 0.001 | 15.80, **< 0.001** |
| Log(Gln) | 64.36, < 0.001 | 231.70, < 0.001 | 38.22, **< 0.001** |
| Log(Asn) | 135.55, < 0.001 | 326.79, < 0.001 | 89.95, **< 0.001** |
| Log(Lys) | 14.39, < 0.001 | 86.61, < 0.001 | 8.95, **< 0.001** |
| Log(Tyr) | 0.91, 0.494 | 45.11, **< 0.001** | 2.14, 0.095 |
| Log(TotalAA) | 43.38, < 0.001 | 154.62, < 0.001 | 25.46, **< 0.001** |
| Log(Glycine/Serine) | 45.30, < 0.001 | 476.15, < 0.001 | 21.34, **< 0.001** |
| Log(Glutamine/Glutamate) | 60.38, < 0.001 | 285.68, < 0.001 | 35.75, **< 0.001** |
| Log(Asparagine/Aspartate) | 106.66, < 0.001 | 440.02, < 0.001 | 62.42, **< 0.001** |
| Log(Gaba) | 5.26, 0.002 | 30.44, < 0.001 | 4.33, **0.006** |
| Log(Aamb) | 1.10, 0.385 | 5.47, **0.028** | 1.13, 0.370 |
| Log(Gsh) | 1.44, 0.245 | 42.85, **< 0.001** | 2.10, 0.101 |

The interaction between line and treatment factors was significant (p < 0.001 less stated) for Gly, Ser, Leu, Iso, Val, Ala (p = 0.019), Phe (p = 0.024), Thr, Arg, Met, Gln, Asn, Lys, TotalAA, Gly/Ser, Gln/Glu, Asn/Asp and Gaba (p = 0.006). Only the main effect of treatment was significant for Tyr (p < 0.001), Aamb (p = 0.028) and Gsh (p < 0.001). Both main effects of line (p = 0.044) and treatment (p = 0.002) were significant, but not the interaction (p = 0.081), for Asp. No significant effects were found for Glu, the grand mean (on the log-scale) for this amino acid being 2.796 with standard error 0.0212.

**Table S2a. Means (on natural log scale) to consider amino acids having a statistically significant (p < 0.05) interaction, along with standard error of the difference (SED) values on 24 df and least significant difference (LSD) values at the 5% level of significance. Lines significantly different (p < 0.05) from WT in each of low and high CO_2_ conditions and with higher quantities than WT are given in bold, with lower quantities in italics.**

| **Amino Acid** | **CO_2_** | **Low** | | | | | |  | **High** | | | | | | **SED** | **LSD (5%)** |
| --- | --- | --- | --- | --- | --- | --- | --- | --- | --- | --- | --- | --- | --- | --- | --- | --- |
|  | **Line** | **33** | **37** | **79** | **84** | **92** | **WT** |  | **33** | **37** | **79** | **84** | **92** | **WT** |  |  |
| Log(Gly) | | **2.660** | **2.622** | **2.305** | **2.898** | **3.012** | 1.804 |  | **0.799** | **0.957** | **0.832** | **1.065** | **0.890** | 0.281 | 0.0974 | 0.2011 |
| Log(Ser) | | **3.277** | **3.355** | **2.011** | **3.414** | **3.190** | 1.554 |  | **1.916** | **1.941** | **1.615** | **2.065** | **1.809** | 1.221 | 0.0780 | 0.1610 |
| Log(Leu) | | **-0.089** | **0.447** | **-1.289** | **0.245** | **-0.311** | -1.821 |  | -1.349 | **-0.904** | **-1.047** | **-1.032** | -1.179 | -1.397 | 0.1072 | 0.2212 |
| Log(Iso) | | **-0.776** | **-0.332** | **-1.660** | **-0.413** | **-0.770** | -3.017 |  | -1.879 | **-1.241** | **-1.408** | **-1.406** | -1.646 | -1.903 | 0.2363 | 0.4877 |
| Log(Val) | | **0.383** | **0.715** | **-0.019** | **0.676** | **0.302** | -0.280 |  | -0.108 | **0.188** | **0.119** | 0.102 | -0.017 | -0.028 | 0.0658 | 0.1359 |
| Log(Ala) | | **1.865** | **1.973** | **1.943** | **2.154** | **1.972** | 1.583 |  | 2.399 | **2.750** | **2.615** | **2.657** | 2.442 | 2.386 | 0.0784 | 0.1618 |
| Log(Phe) | | **0.411** | **0.579** | **0.410** | **0.568** | **0.574** | 0.264 |  | **0.449** | **0.702** | **0.503** | **0.587** | 0.398 | 0.308 | 0.0587 | 0.1212 |
| Log(Thr) | | **2.424** | **2.478** | **1.754** | **2.513** | **2.355** | 1.413 |  | 1.580 | 1.789 | 1.737 | 1.801 | 1.691 | 1.647 | 0.0781 | 0.1613 |
| Log(Arg) | | **0.470** | **0.736** | **0.121** | **0.650** | **0.662** | -0.412 |  | -0.008 | **0.433** | **0.309** | 0.230 | **0.250** | 0.079 | 0.0785 | 0.1620 |
| Log(Met) | | *-1.776* | *-1.737* | **-1.190** | *-1.759* | -1.384 | -1.470 |  | **-1.026** | **-0.848** | **-0.915** | **-0.865** | -1.120 | -1.201 | 0.0803 | 0.1657 |
| Log(Gln) | | **3.854** | **4.137** | **3.271** | **4.130** | **3.612** | 2.862 |  | 2.987 | **3.372** | **3.272** | **3.288** | 3.156 | 3.048 | 0.0735 | 0.1518 |
| Log(Asn) | | **1.648** | **2.047** | **0.415** | **2.032** | **1.284** | 0.001 |  | 0.454 | **0.784** | 0.650 | **0.845** | 0.579 | 0.486 | 0.0820 | 0.1692 |
| Log(Lys) | | **-2.420** | **-0.570** | -5.300 | **-1.690** | **-1.170** | -5.300 |  | -5.300 | -4.350 | -5.300 | -5.300 | -4.970 | -5.300 | 0.6170 | 1.2730 |
| Log(TotalAA) | | **4.965** | **5.145** | **4.498** | **5.182** | **4.928** | 4.179 |  | 4.360 | **4.600** | 4.501 | **4.552** | 4.398 | 4.359 | 0.0698 | 0.1441 |
| Log(Gly/Ser) | | *-0.616* | *-0.733* | 0.294 | *-0.517* | *-0.178* | 0.249 |  | *-1.117* | -0.984 | -0.783 | -1.000 | -0.919 | -0.941 | 0.0794 | 0.1638 |
| Log(Gln/Glu) | | **1.096** | **1.261** | **0.463** | **1.228** | **0.862** | 0.194 |  | 0.173 | **0.538** | **0.439** | **0.456** | **0.518** | 0.202 | 0.0671 | 0.1384 |
| Log(Asn/Asp) | | **-0.681** | **-0.454** | **-1.956** | **-0.489** | **-1.183** | -2.237 |  | -1.943 | **-1.847** | **-1.918** | **-1.669** | **-1.902** | -2.100 | 0.0852 | 0.1759 |
| Log(Gaba) | | **-1.798** | **-1.634** | -2.326 | **-1.690** | **-1.884** | -2.179 |  | -2.293 | -2.046 | -2.198 | -2.288 | -2.244 | -2.240 | 0.1331 | 0.2746 |

**Table S2b. Means (on natural log scale) for amino acids having a statistically significant (p < 0.05) main effect of treatment only, along with standard error of the difference (SED) values on 24 df and least significant difference (LSD) values at the 5% level of significance.**

| **Amino Acid** | **Co_2_** | | **SED** | **LSD (5%)** |
| --- | --- | --- | --- | --- |
|  | **Low** | **High** |  |  |
| Log(Tyr) | -0.660 | -4.720 | 0.6040 | 1.2460 |
| Log(Aamb) | 1.928 | 1.848 | 0.0340 | 0.0702 |
| Log(Gsh) | 4.440 | 4.030 | 0.0626 | 0.1291 |

For Tyr, Aamb and Gsh there was greater and significantly different quantity for the low compared to the high CO_2_ treatment.

**Table S2c. Means (on natural log scale) for amino acid Aspartate, having a statistically significant (p < 0.05) main effect of treatment and of line only, along with standard error of the difference (SED) values on 24 df and least significant difference (LSD) values at the 5% level of significance.**

| **Amino Acid** | **Co_2_** | | **SED** | **LSD (5%)** | **Line** | | | | | | **SED** | **LSD (5%)** |
| --- | --- | --- | --- | --- | --- | --- | --- | --- | --- | --- | --- | --- |
|  | **Low** | **High** |  |  | **33** | **37** | **79** | **84** | **92** | **WT** |  |  |
| Log(Asp) | 2.405 | 2.530 | 0.0360 | 0.0742 | 2.362 | 2.566 | 2.469 | 2.518 | 2.475 | 2.412 | 0.0623 | 0.1286 |

For Asp, there was greater and significantly different (p = 0.044) and quantity for the high CO_2_ treatment. Also, line 37 gave greater and significantly different (p < 0.05) Asp from WT control.

All the transformed lines contained significantly larger quantities of soluble leucine, isoleucine, valine, alanine, phenylalanine, threonine, arginine, glutamine, asparagine and total amino acids than WT under ambient CO_2_ conditions. Lines 37, 79 and 84 contained significantly larger quantities of soluble leucine, isoleucine, alanine and glutamine than WT under high CO_2_ conditions. Lines 37 and 79 had significantly more valine than WT under high CO_2_ conditions. Lines 33, 37, 79 and 84 had significantly more phenylalanine than WT under high CO_2_ conditions. Lines 37, 79 and 92 had significantly more arginine than WT under high CO_2_ conditions. Line 79 had significantly more methionine than WT under ambient CO_2_ conditions. Lines 33, 37 and 84 had significantly less methionine WT under ambient CO_2_ conditions. Lines 33, 37, 79 and 84 had significantly more methionine than WT under high CO_2_ conditions. Lines 37 and 84 had significantly more asparagine than the WT under high CO_2_ conditions. Lines 33, 37, 84 and 92 had significantly more lysine than WT under ambient CO_2_ conditions. Lines 37 and 84 had significantly more total amino acids than WT under high CO_2_ conditions. Lines 33, 37, 84 and 92 had a significantly lower glycine/serine ratio than WT in ambient CO_2_ and line 33 had a significantly lower ratio than WT in high CO_2_ conditions. All lines had significantly higher glutamine/ glutamate and asparagines/aspartate ratio than WT under ambient CO_2_ conditions and lines 37, 79, 84 and 92 had significantly higher ratios than WT under high CO_2_ conditions. Lines 33, 37, 84 and 92 had significantly higher γ-aminobutyric acid than WT under ambient CO_2_ conditions. The content of γ-aminobutyric acid, threonine and lysine was not significantly different in any of the transformed lines when compared to the WT in high CO_2_ conditions.

**Sugars**

The table below shows the statistical significance (p-values) of the main effects of line (WT, 33, 37, 79, 84 and 92) and treatment (low CO_2_ (air) and high CO_2_ (3000 μmol mol^-1^) and the interaction between these two factors.

**Table S3. Significance (F-statistics, p-values) of main effects of line, treatment and the interaction between these factors. F-statistics are on 5 and 24 degrees of freedom (df) for the main effect of line and the interaction, and on 1 and 24 df for the main effect of Treatment. P-values indicating corresponding means tables to investigate are highlighted in bold.**

| **Variable** | **Line** | **Treatment** | **Line.Treatment** |
| --- | --- | --- | --- |
| Log(Glucose) | 4.11, **0.008** | 4.37, **0.047** | 0.84, 0.531 |
| Log(Fructose) | 11.24, **< 0.001** | 43.85, **< 0.001** | 0.50, 0.770 |
| Log(Sucrose) | 9.32, **< 0.001** | 98.72, **< 0.001** | 0.20, 0.957 |
| Log(Total Sugar) | 7.08, **< 0.001** | 22.96, **< 0.001** | 0.51, 0.766 |

For the three particular sugars of interest and the total of these, the main effects means tables are

considered.

**Table S4. Means (on natural log scale) to consider for each particular sugar of interest and the total of these, along with standard error of the difference (SED) values on 24 df and least significant difference (LSD) values at the 5% level of signi**ficance.

| **Sugar** | **Line** | | | | | | **SED** | **LSD (5%)** | **CO_2_** | | **SED** | **LSD (5%)** |
| --- | --- | --- | --- | --- | --- | --- | --- | --- | --- | --- | --- | --- |
|  | **33** | **37** | **79** | **84** | **92** | **WT** |  |  | **Low** | **High** |  |  |
| Log(Glucose) | 3.918 | 3.789 | 3.955 | 3.840 | 3.998 | 4.280 | 0.1205 | 0.2488 | 4.036 | 3.891 | 0.0696 | 0.1436 |
| Log(Fructose) | 3.051 | 2.810 | 2.827 | 2.889 | 2.781 | 3.438 | 0.1057 | 0.2182 | 3.168 | 2.764 | 0.0610 | 0.1260 |
| Log(Sucrose) | 3.751 | 3.710 | 3.649 | 3.718 | 3.686 | 3.894 | 0.0394 | 0.0814 | 3.848 | 3.622 | 0.0228 | 0.0470 |
| Log(Total Sugars) | 4.741 | 4.626 | 4.685 | 4.666 | 4.711 | 5.033 | 0.0781 | 0.1613 | 4.852 | 4.635 | 0.0451 | 0.0931 |

Glucose: All lines significantly different (p < 0.05) from and having lower glucose than WT. Greater accumulation of glucose in low CO_2_ conditions.

Fructose: All lines significantly different (p < 0.05) from and having lower fructose than WT. Greater accumulation of fructose in low CO_2_ conditions.

Sucrose: All lines significantly different (p < 0.05) from and having lower sucrose than WT. Greater accumulation of sucrose in low CO_2_ conditions.

Total sugars: All lines significantly different (p < 0.05) from and having lower total sugars than WT. Greater accumulation of total sugars in low CO_2_ conditions
